# Evaluation of Admixture Silane Added into Cementitious Pastes

**DOI:** 10.3390/ma17225403

**Published:** 2024-11-05

**Authors:** Bruna Aparecida Lamari, Lidiane Fernanda Jochem, Philippe Jean Paul Gleize, Laura Silvestro, Lucas Onghero, Cézar Augusto Casagrande

**Affiliations:** 1 Academic Department of Civil Construction (DACOC), Technological Federal University of Paraná (UTFPR), Curitiba 81280-340, PR, Brazil; lamari@alunos.utfpr.edu.br (B.A.L.); lidiane@utfpr.edu.br (L.F.J.); 2 Department of Civil Engineering (ECV), Federal University of Santa Catarina (UFSC), Florianópolis 88040-970, SC, Brazil; p.gleize@ufsc.br; 3 Civil Engineering Coordination, Federal University of Technology of Paraná (UTFPR), Guarapuava 85053-525, PR, Brazil; laurasilvestro@utfpr.edu.br; 4 Votorantim Cimentos S.A., São Paulo 01448-000, SP, Brazil; onghero.lucas@vcimentos.com

**Keywords:** silanes, VTES, AEAPTMS, MCPTMS, superplasticizer, admixture

## Abstract

This manuscript evaluated the performance of silanes in cementitious matrices in the partial replacement of superplasticizers by silanes. For this, pastes with a water/cement ratio of 0.186 were produced and the superplasticizer admixture based on polycarboxylate esters was partially substituted by three types of silanes—vinyltriethoxysilane silanes (VTES), n-(2-aminoethyl)-3-aminopropyltrimethoxysilane (AEAPTMS), and methacryloxypropyltrimethox-ysilane (MCPTMS)—in two substitutions levels (20% and 40%), and then tested in Portland cement pastes. Specific gravity, trapped air, mini-slump, and hydration kinetics (evaluated by isothermal calorimetry up to 48 h) of the pastes were determined in the fresh state. Thus, in the hardened state, the compressive and flexural strength tests (7 and 28 days), specific gravity, and absorption by immersion of the pastes were carried out. The results showed that the substitution of 20% and 40% of VTES and AEAPTMS considerably reduced the workability and increased the air content of the pastes in comparison to the reference sample. In contrast, the incorporation of 20% and 40% of MCPTMS did not significantly affect these properties. The presence of silane, for all analyzed samples, had a delaying effect on the hydration process: the maximum delay verified had a hydration peak in approximately 36 h for the 40% MCPTMS sample and the minimum delay verified had a hydration peak in approximately 11 h for the 20% VTES sample. The replacement of 20% and 40% by any of the silanes progressively reduced the flexural strength at both 7 and 28 days. In the compressive strength, as well as in the tensile strength in flexion, there was a decrease in the results when compared to the reference, except for the MCPTMS, at 7 and 28 days. In immersion absorption, all samples showed high percentages of absorption and void index when compared to the reference.

## 1. Introduction

Silane is one of the main silicon-based reagents that is still little explored by the construction industry, but it has great potential to improve the workability and increase the mechanical strength of cement–matrix composites [1,2,3].

As a chemical reagent consisting mainly of silicon atoms as the main element and covalently bonded to hydrogen atoms, it normally presents two types of reactivity in the same molecule: one polar and the other nonpolar. These molecules are referenced by a hydrolyzable group, for example, alkoxy ($C_{x}H_{y}O$), and a functional group, for example, amine (-${NH}_{2}$) and vinyl (−C=C) [4,5,6]. Silanes have a small molecular skeleton, with a diameter of 1.0 × 10^−6^ a 1.5 × 10^−6^ mm, are water-repellent, colorless, and are found in their chemical form as alkoxysilane. Given that they are small, they are very volatile, aiding in the effectiveness of penetration even in a denser substrate [7].

During the year 1940, the efficiency of silane as a binding agent was confirmed when combined with polyester composites reinforced with fiberglass [8]. Thus, in the following century Gelest [9] disclosed in its catalog that there are more than 420 different types of silanes, which are used to increase the mechanical properties (flexion and compression) of composites, improve adhesion, increase the dispersion of pigments and minerals, immobilize catalysts, and bind biomaterials.

Silane is classified according to the molecules and radicals of greatest interest, namely alkylsilanes, silanols, and silanes. Alkylsilanes correspond to alkanes: that is, a carbon linked to four alkoxy groups, but with the carbon atom replaced by silicon atoms. Silanols are silane derivatives in which a hydroxyl is linked to a silicon atom, presenting greater reactivity with silane particles. Siloxanes are characterized by the bond between silicon and oxygen, very similar to ether [10]. Silanes are initially named by the acronym of the organofunctional group, followed by the intermediate group linked, if any, to silicon, followed by the acronym of the alkoxy groups: for example, intermediate–functional group-alkoxysilane [2]. The composition of the silane structure has two different reactive centers: one contains alkoxy groups that hydrolyze and form silanol groups that are capable of condensing or reacting when there is the presence of kaolin or silica, and the other contains amino, epoxy, vinyl, or mercapto groups, which react with the polymer matrix [11].

According to Plueddemman [12], covalent bonds can occur between the coupled surface and the silanol groups (Si-OH). This procedure is called the coupling mechanism, which can be divided into four parts: the formation of the bond between the inorganic substrate and the silane, in the case of silicates from the hydration of cement, and silica with cementitious materials [13]. Thus, with the hydrolysis of the three hydrolyzable groups, the condensation stage occurs, where the formation of siloxane oligomers occurs, which binds to the OH^−^ groups of the substrates through hydrogen bonds. However, the hydrogen bonds are transformed into covalent bonds with the release of water molecules, resulting in curing or drying. Although these reactions have been described sequentially, they can also occur simultaneously. Thus, the chemical reaction of the silane component depends on the substrate with which it is associated [4,14,15,16,17,18]. The effectiveness of the chemical bond between the silane and the inorganic substrate silica, present in Portland cement, is considered excellent.

The amine and alkoxide groups have highly polar aminosilane molecules that exhibit strong chemical interactions with cementitious matrices. Svegl, Suput-Strupi, and Skrlep [19] evaluated the influence of aminosilanes on the macroscopic properties of cement paste. The authors found that the effect of APTES and AEAPTES silanes on fresh cement paste and mortar properties increases workability, decreases the water/cement (w/c) ratio, and slows down the hydration process. In the hardened cement paste and mortar properties, they increase compressive strength after 28 days of curing (5% compared to the reference) and increase flexural strength after 28 days of curing (20% compared to the reference).

The partial use of the compound N-[3-(trimethoxysilyl)propyl]aniline (TMSPA) can accelerate hydration and improve pore structures in hardened cement paste; the mechanical strength becomes higher at 3 days of age due to these factors, according to He, Zhang, and Hooton [20], in the study on polycarboxylate-based superplasticizers in which the acrylic acid compound was partially replaced by 3-(trimethoxysilyl) propylmethacrylate (TMSPMA).

The partial addition of amino-, vinyl-, and epoxy-based silanes to common mortars presents a significant improvement in flexural and compressive strength at the ages of 7 days and 28 days by adding the silane reagent. Thus, when epoxy-based silane is added to high-strength mortars, there is a noticeable increase in compressive strength (30%), tensile strength (38%), and modulus of elasticity (13%) compared to the control mortar, as found by Feng et al. ([21]), on the effects of silanes and silane derivatives on cement hydration and mechanical properties of mortars. The improvement in the workability and mechanical strength of concrete using silane-treated microsilica was evaluated by Xu and Chung [22,23], who found that the workability of mortars increases due to the intermolecular interaction of microsilica with water. Strengthening the bond between silica and cement generates an increase in tensile strength of 31% and compressive strength of 27%, in addition to a significant increase in flexural strength, moisture content, and specific gravity.

This study evaluated three types of silanes (amino-, vinyl-, and acrylic-based) in their interaction with a superplasticizer additive in the cement matrix. The objective of this study was to compare the replacement of the superplasticizer additive by three types of silanes in different percentages (20% and 40%) used in the production of Portland cement pastes. In this study, properties were evaluated in the fresh state, such as workability, apparent specific gravity, and isobaric calorimetry, and in the hardened state, with properties such as flexural and compressive strength, in addition to qualitative analyses of fractographies.

## 2. Experimental Section

### 2.1. Materials

For the production of cement pastes, a Brazilian Portland cement (CP V) [24] with a high initial strength, equivalent to the Type III by ASTM C150 [25] standard, was used. This cement was selected because it has a high clinker content (>90%), minimizing the side effects of other mineral additions. Table 1 presents the chemical and mineralogical composition of the cement, and Table 2 presents the main physical characteristics of Portland cement as indicated by the manufacturer.

Three types of silanes were used to produce the cementitious pastes: one is called vinyltriethoxysilane (VTES), which has a vinyl functional group at one end of the molecule and three hydrolyzable ethoxy groups at the other end; another silane is called n-(2-aminoethyl)-3-aminopropyltrimethoxysilane (AEAPTMS), which has an amino-type functional group at one end and three hydrolyzable methoxy groups at the other end of the molecule; and there is a methacryloxypropyltrimethoxysilane (MCPTMS) silane, which has an acrylic-based functional group at one end and three hydrolyzable methoxy groups at the other end. Each type of silane was used as a partial replacement for the superplasticizer admixture in portions of 20 and 40% (by mass) to produce the cementitious pastes. The main characteristics of the silanes used are presented in Table 3.

The chemical admixture, also called the superplasticizer, used in this study is a third-generation superplasticizer function, based on a modified polycarboxylic ether chain that acts as a steric and electrostatic on the cement surface. This admixture complies with NBR 11768-1 [26] and ASTM C494 [27]. The solids content of the superplasticizer is 40%.

### 2.2. Methods

The pastes were produced with a water/cement ratio of 0.186 and a 1% water-reducing admixture (superplasticizer) in relation to the weight of the cement. For samples with silanes, the superplasticizer was partially replaced by one of the silanes in the fraction of 20% and 40%, making a total of 1% in relation to the weight of cement, as a reference. The sample compositions are presented in Table 4.

Kantro’s method [28] was used to evaluate the effect of the admixture and silane modifications on cement. This method involves filling a mini frustum-conical mold with Portland cement paste on a horizontally leveled glass plate. After the elevation of the mini-slump and consequent spreading of the mixture, the average of the three measured diameters was calculated, thus obtaining the equivalent spreading ([29,30,31,32]).

NBR 13278 [33] was used to determine the specific gravity in the fresh state. Entrapped air can be defined as the sum of air that was not eliminated during the densification process of the paste and concrete. The apparent specific gravity test allows the determination of the air content trapped in the paste based on the apparent specific gravity of the evaluated paste and the theoretical specific gravity without air. This test was carried out in accordance with NBR 9833 [34].

To evaluate the hydration kinetics of cement, the adiabatic calorimetry test was performed, in accordance with ASTM C1753 [35]. In this process, heat is released, resulting from the hydration of the cement from the first moments of contact with the mixing water. In this work, a data collector was used that recorded data every 2 s, and a type K thermocouple was used as the measurement method [36]. The induction period is referred to as the period of lowest cement hydration activity until the beginning of the acceleration period. There are interpretations about when the induction period ends and how it can be calculated [37,38,39,40,41,42,43,44]: in this work the end of the induction period was determined when the cement began to set, which was calculated from the second derivative of the heat of the hydration graphical function [45,46]. The cement setting time is referred to as the period in which the material presents the transition from plastic to rigid and also has some interpretations about when it ends [47,48]. Therefore, in this work the end of setting was indicated as the highest value obtained by the first derivative of the hydration heat curve. To calculate the normalized temperature, Equation (1) was used, where $T_{n}$ is the normalized temperature in °C·g of cement^−1^, T is sample temperature, $T_{o}$ is the empty crucible used as reference sample of the environment, and $c_{mass}$ is the cement mass of the sample.

(1) $$T_{n}=\frac{T-T_{o}}{c_{mass}}$$

To determine the flexural strength, the three-point bending test was performed, in accordance with NBR 13279 [49], using test specimens measuring 40 × 40 × 160 mm. At least 3 specimens were used for each series of pastes. Once the flexural strength was determined, the compressive strength of the samples was determined, according to NBR 13279 [49], where the two halves of the flexural strength test specimens were tested in compression, in an area of 40 × 40 mm. At least 6 specimens from each series were tested.

To determine the apparent specific gravity in the hardened state, according to NBR 13280 [50], 3 test specimens measuring 40 × 40 × 160 mm were used for 28 days of hydration, per series. The water absorption test by immersion was carried out, in accordance with NBR 9778 [51], which determines how much water the test specimen can absorb in relation to its initial mass.

## 3. Results and Discussion

### 3.1. Fresh State Results

**Figure 1:**
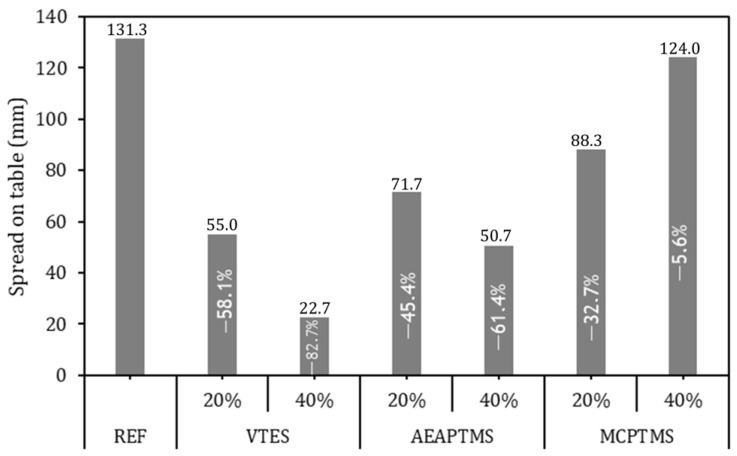
Workability results of cement pastes. Sample values are presented at the top of the bars and the percentage of variation relative to the REF series is presented in the middle of the bars in white text.

Figure 1 presents the results of the spread on the table obtained by mini-slump using Kantro’s method. The REF series obtained an average spread of 131.3 mm and all series with silane addition showed a lower workability; however, no general trend was observed for the workability of the pastes. The VTES 20% series obtained 55.0 mm, which is a 58% less spread compared to the REF series. The VTES 40% series obtained 22.7 mm, which is an 83% less spread compared to the REF series. The APTMS series obtained a 45% and 61% less spread for the 20% and 40% series, respectively, compared to the REF series. The MCPTMS 20% series obtained 88.3 mm, which means a 32.7% less spread than the REF series. Thus, the MCPTMS 40% series obtained 124 mm, which means a 5.6% less spread than the REF series.

It was expected that the behavior of pastes with a lower amount of superplasticizer admixture would present a lower workability [52], associating with a lower effectiveness of silane in the fresh state (VTES and AEPTMS samples), which is in accordance with [53]. This could be justified by the lower hydrolysis rate of the silanes and consequently the lower ionization capacity of the molecule, which would result in a lower efficiency in general. On the other hand, the samples with MCPTMS had a different behavior, where the greater presence of silane resulted in a greater workability for these series with silanes, which is in line with [54]. This may be associated with the nature of silane, which tends to be more electronegative and, therefore, more reactive, mainly due to its greater solubility and higher hydrolysis speed [17,18], which has a great influence on the ionization of the molecule and consequently on its efficiency in the cement paste [23]. According to the ANOVA performed, with 95% confidence, it is possible to state that the average value of the samples is different from the REF series (*p*-value < 0.05), indicating that the silanes had an impact on the spreading of the cement paste studied.

**Figure 2:**
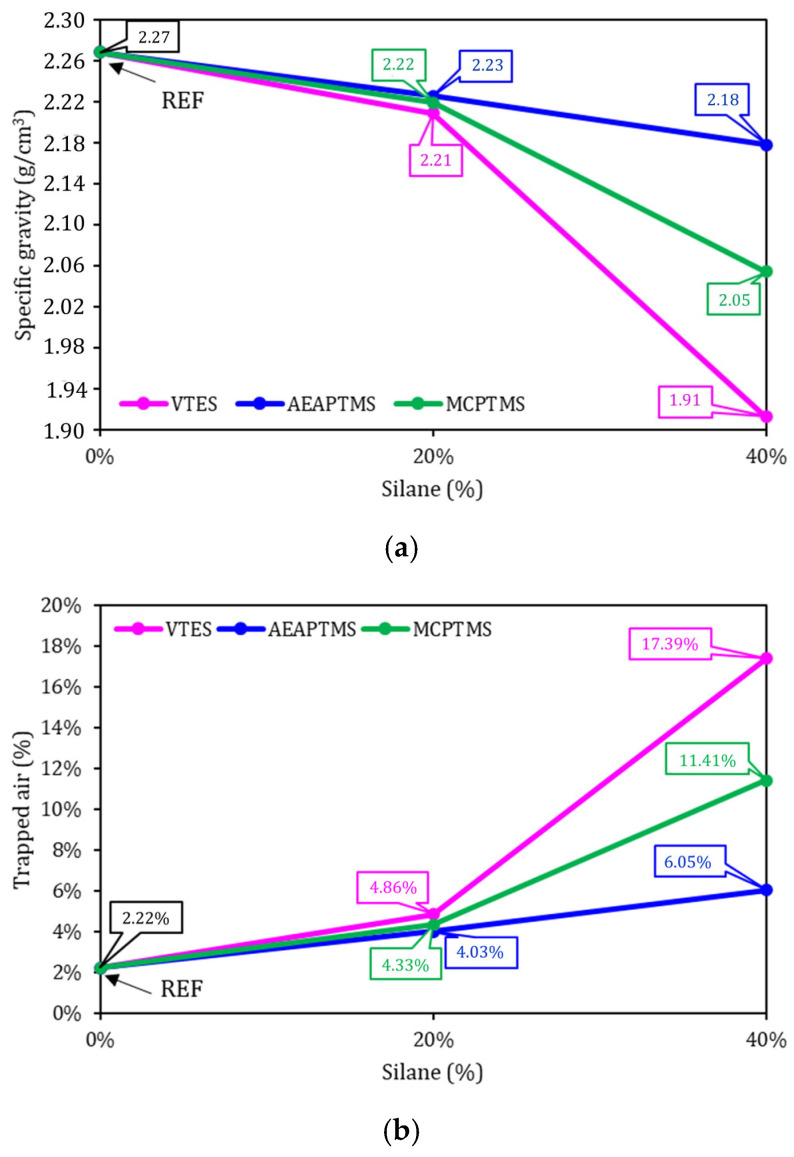
Physical properties results. Specific gravity in (**a**) and trapped air in (**b**).

Figure 2 shows the results of the physical properties of cement pastes. Figure 2a shows the apparent specific gravity and Figure 2b shows the result of trapped air. It was verified that the REF sample presented 2.27 g·cm^−3^ and the other samples with the presence of silane presented a lower specific gravity than the REF series. In this test, a logical trend occurred in all samples with silane, where the greater the amount of silane, the lower the specific gravity value, which is reflected in the trapped air content, which showed the opposite trend—that is, the greater the amount of silane, the greater the amount of trapped air—which is in line with the workability results (Figure 1). With a lower workability, the mixture in the fresh state becomes more rigid and viscous, resulting in greater difficulty in releasing air during molding and compaction, as reflected in the lower specific gravity value and higher air value in the sample. The silane molecule is initially not soluble in water and does not present immediate reactivity with cementitious compounds after its addition, as occurs with superplasticizer admixture. Therefore, the reduction in the specific gravity of pastes with an increasing silane content may be derived from their nonpolar nature [55]. The effect on specific gravity is low [21] (less than 3% for the series with 20% silane added), with the largest variations found being 0.35 g·cm^−3^ compared to REF. On the other hand, the effect on the trapped air content is significant [23,56], so that the series with 20% silane obtained in the order of 80–120% greater entrapped air compared to the REF series, while the series with 40% silane presented 200–780% greater air content compared to that obtained by the reference sample.

The ANOVA performed, with 95% confidence, indicated that the apparent specific gravity values of the samples with 20% silane are comparable to the REF sample. However, for 40% silane there were greater variations, and these results were considered statistically different compared to the reference series. The replacement of 20% and 40% of the superplasticizer by silane, in all cases, increased the air content of the pastes [19,57], and this increase is statistically significant, meaning that silane influences the air trapped in the cement pastes.

**Figure 3:**
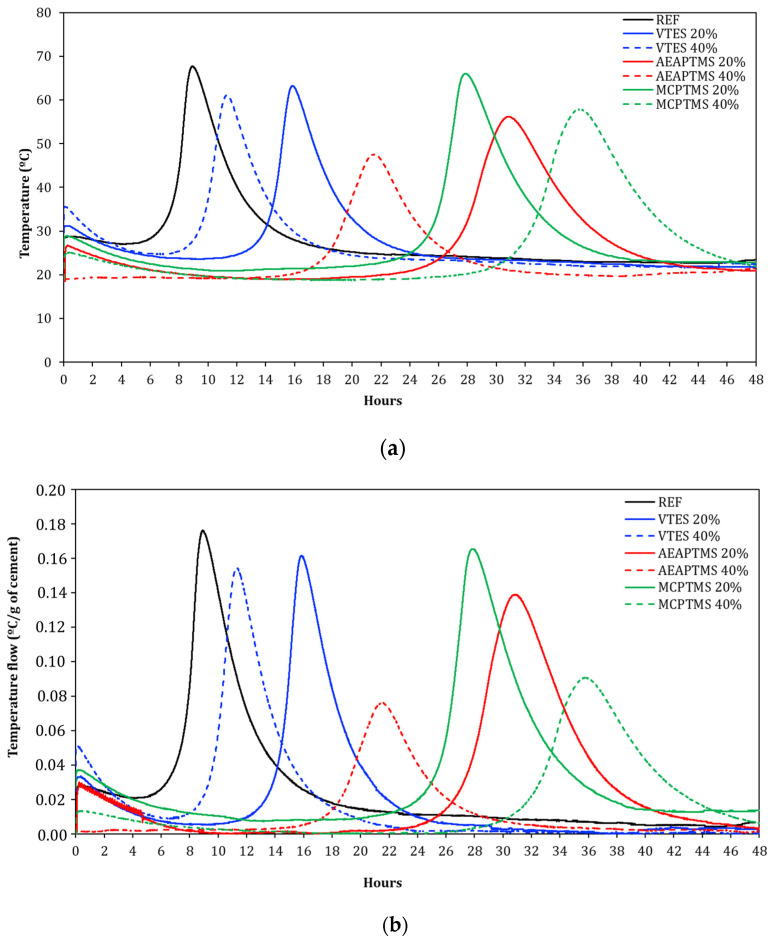
Results of the adiabatic calorimetric. Temperature variation in (**a**) and normalized temperature variation in (**b**).

Figure 3 shows the temperature variation curves for cement hydration as a function of time during the hydration process of the pastes over the 48 h test period. Figure 3a shows the temperature variation obtained in the test and Figure 3b shows the normalized temperature variation, which is the difference between the temperature obtained and the reference temperature divided by the amount of cement in the paste [58]. The heat of hydration refers to the heat released when water reacts with cement to form hydrated compounds, the main one being calcium silicate hydrates (C-S-H), which contribute to the development and strength of the composite [32]. The heat of hydration is an important parameter in concrete construction, as it influences several factors, such as curing, thermal cracking, and overall material performance [40].

Initially, immediately after the addition of water, there is a rapid increase in the heat of hydration, the initial heat peak, caused by the exothermic reactions between the aluminates and water. After this, the heat release gradually decreases over time as the hydration reactions progress slowly: this period is called the dormant or induction period, and the duration varies depending on the type of compound and the amount used. Subsequently, a period of accelerated heat evolution occurs—which is the main heat peak, called the acceleration peak—derived from the ongoing hydration reactions mainly of tricalcium silicate (C_3_S). After the main heat peak, the rate begins to decrease, entering the deceleration phase, with a decrease in hydration reactions and an overall reduction in the heat of hydration [59].

The REF sample presented a hydration acceleration peak at a temperature of 67.7 °C. In the VTES 20% and 40% samples, the acceleration peak presented temperatures of 60.4 and 62.2 °C, respectively. In the AEAPTMS 20% and 40% samples, the acceleration peak presented temperatures of 55.5 and 47.0 °C, respectively, and in the MCPTMS samples with a dosage of 20% and 40%, the acceleration peak presented temperatures of 65.5 and 57.5 °C, respectively. In general, for all the samples analyzed, the most significant effect is the retardation effect of the acceleration peak, compared to REF. Furthermore, an inverse relationship was observed between the percentage of silane incorporation and the hydration acceleration peak. The presence of silanes also significantly influenced the duration of the induction period, which is associated with the presence of ettringite, precipitation of portlandite (Ca(OH)_2_), and formation of C-S-H [38,60,61].

**Figure 4:**
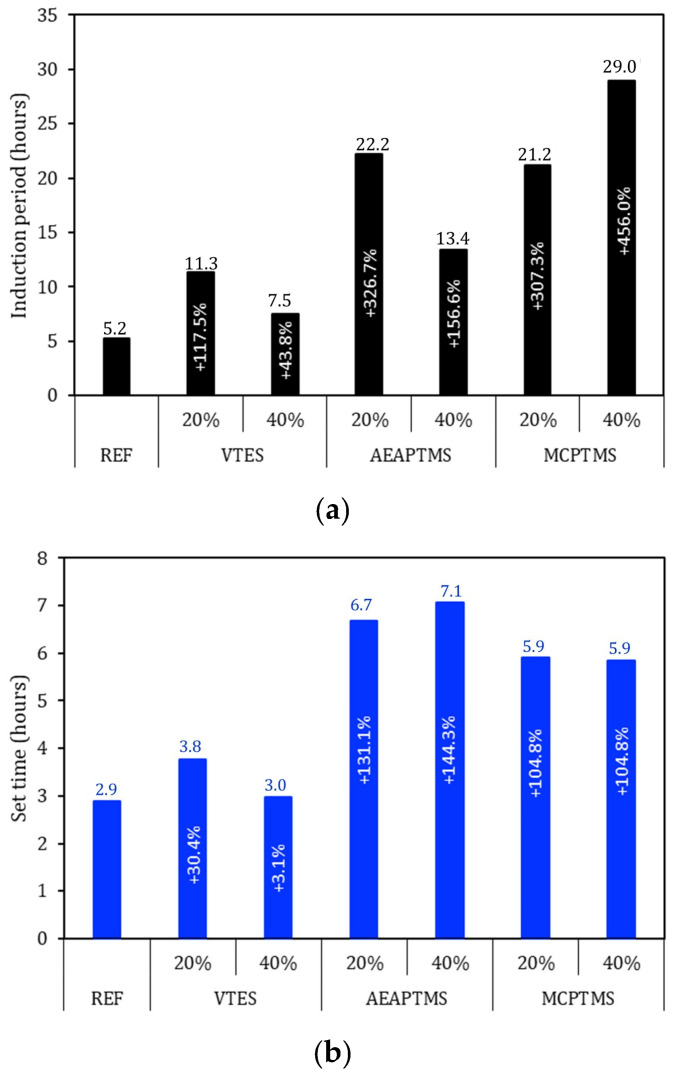
Evaluation of the initial part of cement hydration. Induction period in (**a**) and set time in (**b**). Sample values are presented at the top of the bars and the percentage variation compared to the REF series is presented in the middle of the bars in white text.

The induction period of Portland cement is a phase in the hydration process, also known as the dormancy phase, which occurs after the cement has started to mix with water. During this period, the chemical reaction between the cement (mainly C_3_S compounds) and water slows down significantly, allowing the material to remain workable enough to be applied. This induction period usually lasts 2–4 h. After the end of the induction period, cement hydration is accelerated by the saturation of the solution with Ca(OH)_2_, leading to the beginning of setting and, subsequently, to an increase in strength. Controlling the induction period is critical in construction material engineering, as it directly affects the workability time of concrete and the initial development of its strength. This period depends on several factors, such as the chemical composition of the cement, the fineness of the particles, the presence of additives, and environmental conditions. Figure 4a shows the results of the first part of the cement hydration, until the end of setting, characterizing the cement setting time. It is possible to see that regardless of the silane used, the induction period was longer. There were increases in the order of 43.8% to 456% in relation to the REF. This factor is associated with the ability of the modified additive to interact with the cement more effectively than the original superplasticizer alone [53,62]. It is noted that the larger the functional group present in the silane, the greater the change in the induction period. This may be associated with the ability of silane to interact with Ca(OH)_2_ in the solution, delaying the precipitation of this compound. That is, the silane-modified superplasticizer admixture interacts at one end with the cement particle and at the other end with Portlandite, resulting in the observed effect. There is still a controversial fact, as the expectation was that the greater the amount of silane, the greater the impact, as observed for the MCPTMS samples. However, samples with VTES and AEAPTMS with greater amounts of silane showed a lesser effect on the induction period (compared with the 20% and 40% samples). This factor may be associated with the silane’s ability to interact with neighboring silanes after the hydrolysis of the alkoxy groups, thus losing part of its ability to react with the cement surfaces or with Ca(OH)_2_, resulting in the greater impact of the 20% samples compared to the 40% samples [54]: for these types of silanes, this is an indication of their saturation point.

Figure 4b shows the results of the cement setting time. As observed for the induction period, all samples with the presence of silanes showed a longer cement setting time, which means that the cement is taking longer to become rigid [63]. There were variations compared to the reference, from 3.1% (VTES 40%) to 144% (AEAPTMS 40%). This behavior is a consequence of the interaction of the silane with Ca(OH)_2_ during the induction period, which needs to neutralize the silane so that it can then be available in solution to subsequently precipitate. This takes time, so much so that the setting time of the cement is significantly extended. In this analyzed parameter, the silanes with a larger molecular chain size in the functional group also presented a longer setting time, with the AEAPTMS silane having the greatest impact on this parameter.

**Figure 5:**
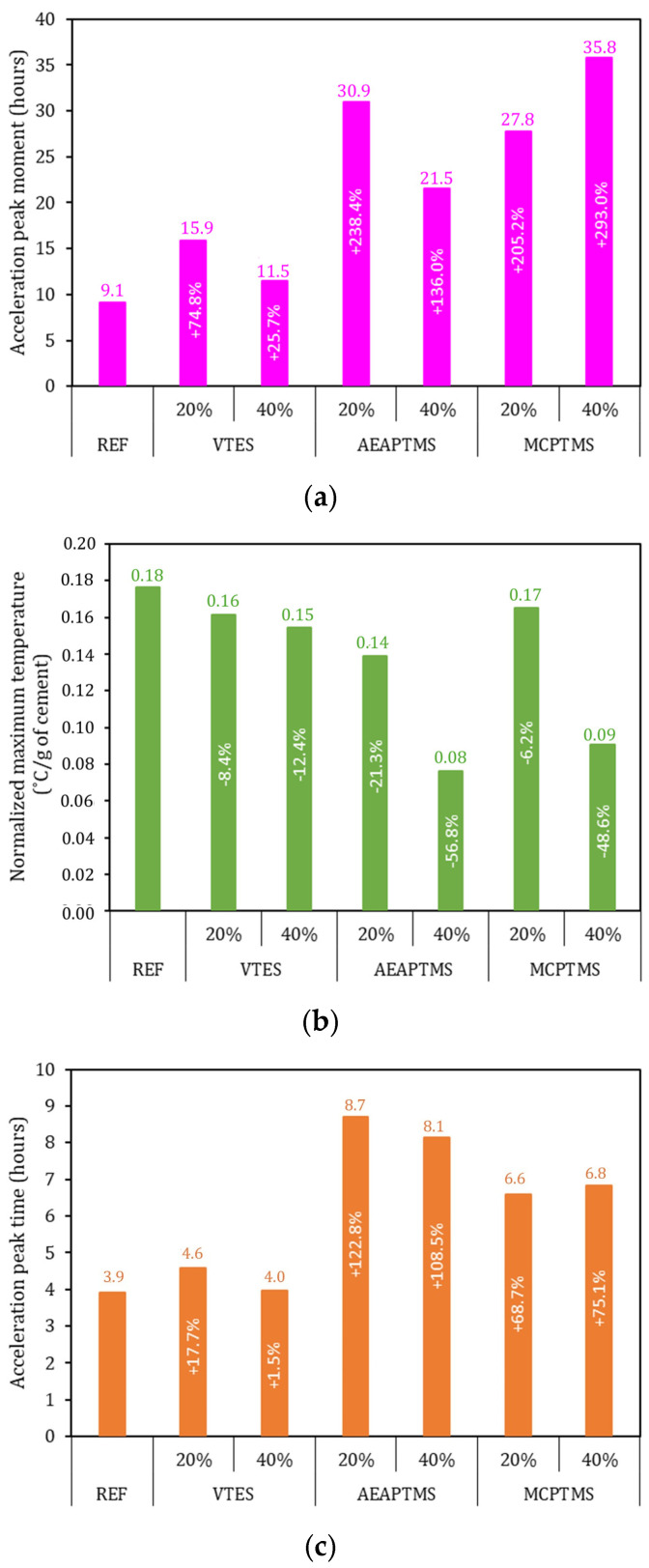
Evaluation of the acceleration peak of cement hydration. Moment of peak acceleration in (**a**), maximum normalized temperature in (**b**), and peak acceleration in (**c**). Sample values are presented at the top of the bars and the percentage of variation relative to the REF series is presented in the middle of the bars in white text.

Figure 5 shows the results of the peak acceleration of cement hydration. Figure 5a shows the moment when the peak acceleration reaches its maximum value in the test. The REF showed the maximum value after 9.1 h of mixing the cement with water. For all samples, a significant and pronounced increase was observed for this analyzed parameter, reaching values up to 295% higher than REF [21]. This was due to the delay in hydration, verified during the prolonged induction period in the samples with the presence of silane, as shown in Figure 4a. Figure 5b shows the results of the maximum value of the normalized temperature. In sample REF, 0.18 °C/g of cement was obtained, while in all samples with the presence of silane there was a reduction in this parameter. Samples with the presence of VTES presented approximately 10% less heat, while samples with the presence of AEAPTMS presented from −21% to −56% less heat than the REF sample. Samples with the presence of MCPTMS presented −6% to −48% less heat compared to the REF sample. This may be associated with the delay hydration of samples with the presence of silane. Although the induction period is a period of low chemical activity of the cement, there is still a small amount reacting slowly and therefore releasing some heat. Thus, part of the energy that should have been released in the acceleration period had already been released due to the extension of the induction period. This hypothesis becomes more evident when evaluating Figure 5c, where all samples with the presence of silane presented a time associated with the acceleration peak that was greater than the reference. The REF sample presented 3.9 h releasing heat in the hydration acceleration, while the samples with silane presented values of up to 8.2 h (122% higher). This means that the samples with silane had the reactions associated with C_3_S delayed and prolonged, resulting in a less intense but more prolonged peak. When VTES and AEAPTMS silanes are added to the system, they interact with the Ca^2+^ and OH^-^ ions present in the solution. They also interact with the cement and the surface of C-S-H, which is the main component of cementitious materials. These interactions between silanes and the various components can have an impact on the hydration process. One observed effect is a decrease in the degree of saturation of C-S-H and Ca(OH)_2_ in the aqueous phase. In other words, the amount of C-S-H and Portlandite that can be formed and dissolved in water is reduced. This phenomenon has been reported by several studies [21,54,55]. These studies observed that as the amount of silane increases, there is a corresponding decrease in the saturation level of C-S-H and Portlandite in the aqueous phase. This suggests that silanes influence the formation and stability of these compounds and may affect the overall hydration process and resulting properties of cementitious materials.

### 3.2. Hardened State

**Figure 6:**
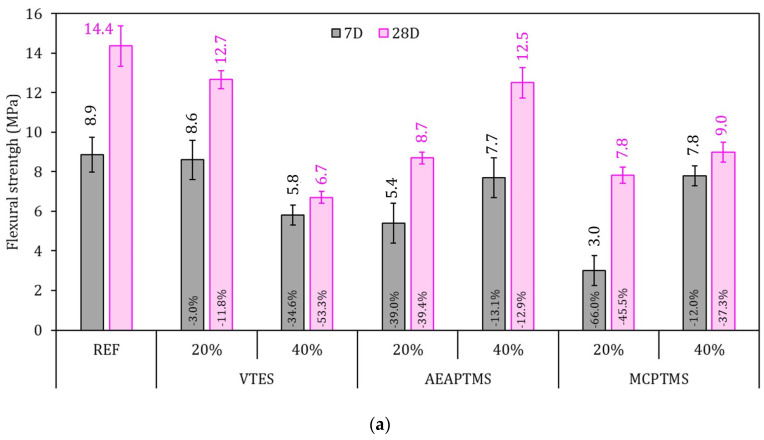

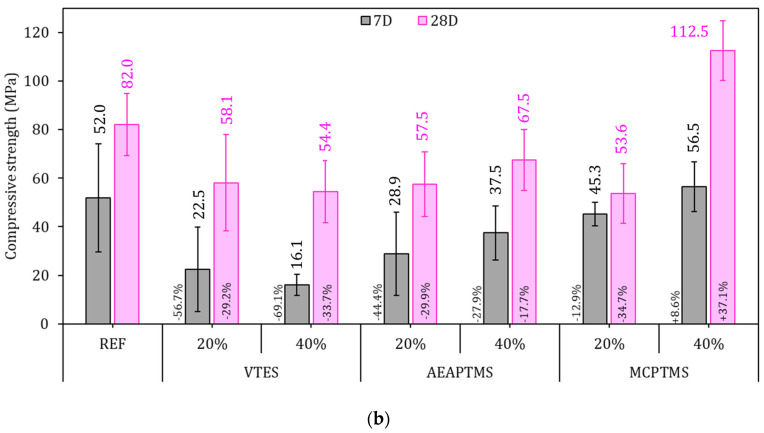
Hardened strength results. Flexural strength in (**a**) and compressive strength in (**b**). The error bars stand ± 1 standard deviation. The sample values are presented at the top of the bars and percentage of variation in comparison to the REF series is presented in the middle of the bars in black text.

In Figure 6, the hardened strength results for the pastes are presented. The samples were tested at 7 and 28 days of cement hydration. The flexural tensile strength (Figure 6a) of the reference sample for 7 and 28D reached 8.9 MPa and 14.4 MPa, respectively, while the flexural strength of the samples with the presence of silane presented lower results than the REF sample in all the analyzed variations, both for 7 and 28D of hydration. For 7 days, the reduction in the samples with silanes varied from −3% to −66%, while at 28D the variation was from −11% to −53% compared to REF. This may be associated with the change in the hydration kinetics of the samples. It was found that the heat flux of the samples with silanes was significantly different from the REF sample, mainly by verifying that the acceleration peak was reduced in its intensity; this may be associated with a lower solubilization of C_3_S and a lower formation of C-S-H, which will result mainly in a lower strength at early ages, in this case at 7D [64]. At 28 days, the retarding effect of silane tends to be lower on strength, since the hydration reactions are mainly associated with the dissolution of C_2_S and the silane has already been degraded within the cement matrix [37,65]. This can also be explained by the results presented in the property tests in the fresh state: the pastes presented losses in workability and high levels of trapped air compared to the reference. Therefore, it is directly related to the loss in mechanical properties [19].

Figure 6b shows the compressive strength results of the cement pastes. The REF sample obtained 52.6 MPa and 82.1 MPa for 7 and 28D of cement hydration, respectively. It can be seen that the presence of silane significantly affected the compressive strength of the samples. With the exception for the 40% MCPTMS sample, all samples with silanes presented a lower strength than the reference sample, both at 7D and 28D of cement hydration. This may be associated with the delay in hydration observed in the calorimeter tests. As there is a delay in hydration, there is less C-S-H precipitated in the same period compared to the REF sample; therefore, the expectation is that there will be an impact on the mechanical strength of the composite [66]. The MCPTMS 40% sample showed an 8.6% increase in compressive strength at 7D and a 37.1% increase at 28D of cement hydration compared to the reference sample.

**Figure 7:**
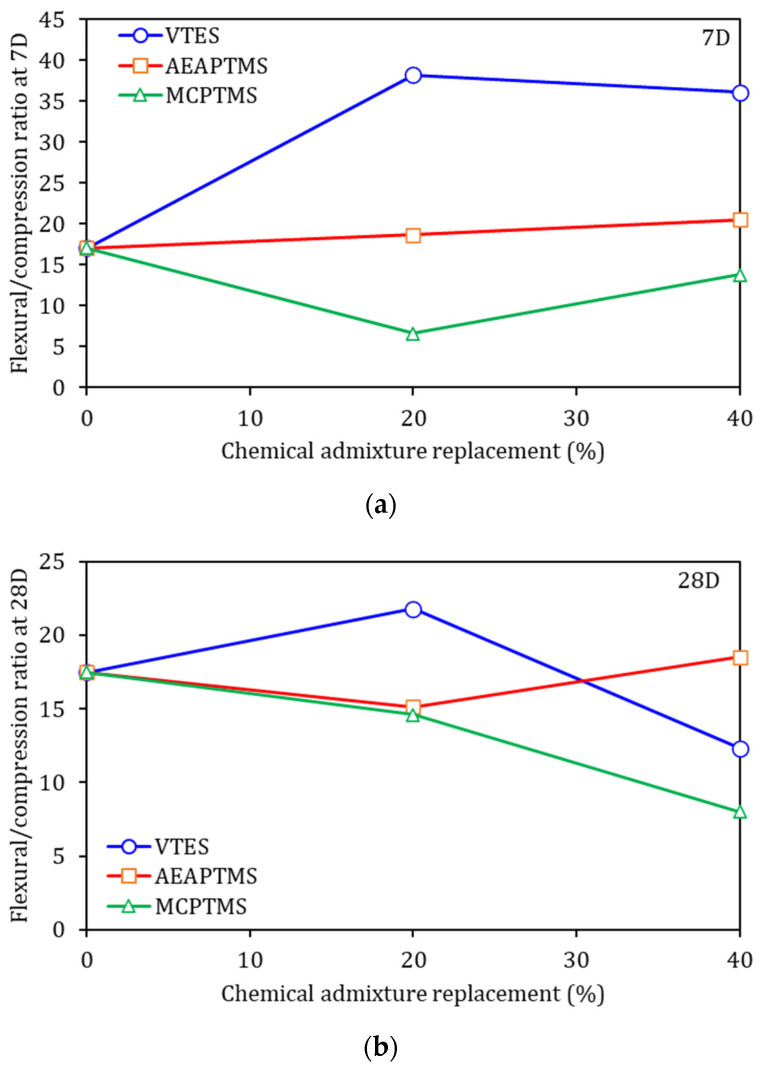
Flexural-to-compression ratio, at 7D in (**a**) and at 28D in (**b**).

When a ceramic material is subjected to flexural tensile testing, the stress is concentrated at the ends of these cracks, increasing the probability of propagation and growth, resulting in poor mechanical behavior, as expected for a defect-free material (GRIFFITH, [67]). Furthermore, measuring the toughness of ceramic composites is hampered by their brittle elastic behavior, which means a low capacity to resist crack propagation. Thus, one way to evaluate the toughness of ceramic composites can be through their flexural strength/compressive strength ratio [53,54]. Figure 7 shows the flexural strength/compressive strength ratio of the samples at 7D and 28D of cement hydration. In comparison with the reference, the higher the value, the tougher the sample can be considered. Thus, at 7D there is no trend identified for the samples with the presence of silane (Figure 7a). For one type of silane (VTES), there was an improvement in the indices, while for the AEAPTMS samples there were indices comparable to the REF, and for the sample with MCPTMS there were results lower than the reference. This is due to the variability in the flexure test, mainly in the first days of hydration.

In Figure 7b, only the VTES 20% sample obtained higher toughness indexes than the REF sample, while all the other samples presented lower toughness indexes than the REF sample. In general, it can be said that the toughness did not show any improvement when silane was used in the composite composition. This can be mainly associated with the trapped air, which has a greater influence on the flexural strength results, thus decreasing the ratio [54].

**Figure 8:**
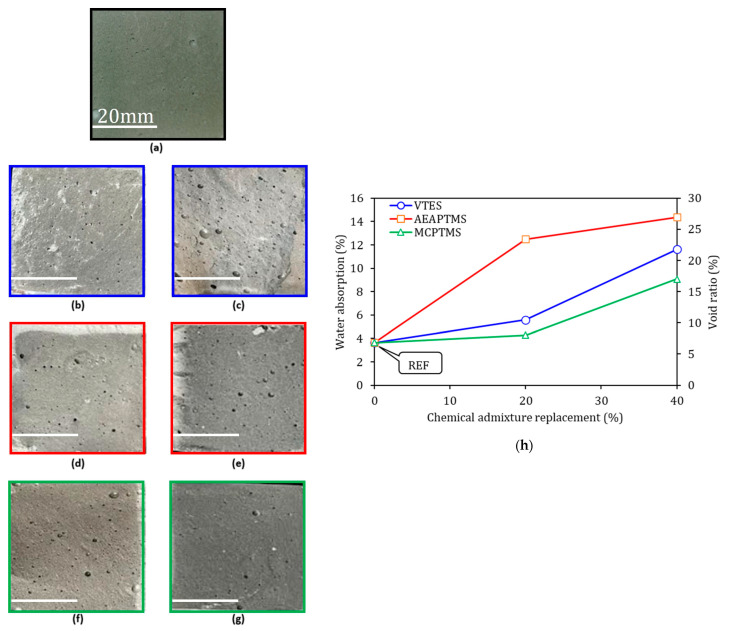
Fracture surface of cement pastes in 7D. REF in (**a**); VTES 20% in (**b**); VTES 40% in (**c**); AEAPTMS 20% in (**d**); AEAPTMS 40% in (**e**); MCPTMS 20% in (**f**); MCPTMS 40% in (**g**); and water absorption and void index in (**h**). The photos of the fracture surface were obtained with an optical cell phone with a 26 mm lens.

Figure 8 shows the fractographies of the pastes at 7D of cement hydration, water absorption, and void content. The fracture surfaces were derived from the samples tested under flexure.

The REF sample Figure 8a showed small trapped air bubbles, similar to those observed in the samples with 20% VTES and MCPTMS, Figure 8b,f. The samples with 40% silane showed a pronounced porosity, as verified in Figure 8c,e,g, which is in line with the water absorption and void index tests in Figure 8h. The effect of silane in the mixture is evident, as these samples have 40% substitution in their composition, and the large trapped air bubbles that can be observed on the fracture surface are noticeable, justifying the lower mechanical performance and specific gravity of these samples.

The water absorption and void index tests are associated with the total porosity of the sample in the hardened state. The water absorption of the REF sample was 3.63%. The water absorption for the VTES and MCPTMS samples with 20% had similar values, being 5.58% and 4.27%, respectively (statistically equal to REF according to the t-student). For the AEAPTMS samples 20% and 40%, the greatest increases in absorption were found, being 244% and 296% in relation to the reference, respectively. For the VTES and MCPTMS samples in the amount of 40%, increases of 220% and 150%, respectively, were also obtained in relation to the reference. These absorption and void results can be justified by the porosity observed in the fractographies, results that evidence the effect of silane on the porosity of the studied samples.

## 4. Conclusions

This study evaluated the changes in the fresh and hardened properties of cement pastes with the partial replacement of the superplasticizer by VTES, AEAPTMS, and MCPTMS silanes. Based on the results obtained, the following conclusions can be drawn:

- The partial replacement of superplasticizers by silanes (VTES and AEAPTMS) resulted in a lower workability and higher entrapped air content in the pastes, which contributed to the reduction in apparent density and an increased porosity. This suggests that silanes affect the behavior of concrete in the fresh state, mainly at the higher proportions studied (40%).
- The presence of silanes delayed the cement hydration process compared to the reference sample. This delay was more pronounced in the samples with MCPTMS, with hydration delays up to 295% greater than the reference. This effect suggests that silanes affect the hydration kinetics, prolonging the induction period and the formation of hydration products.
- When MCPTMS was used, there was a tendency for an increase in compressive strength at 7 days and 28 days, while when VTES or AEAPTMS was used, there was a tendency for these properties to decrease regardless of the age of the test, mainly for the 40% replacement. These effects can be attributed to the trapped air verified in the fresh state and confirmed by the fracture surface images.

## Figures and Tables

**Table 1:** Chemical and mineralogical composition of the Portland cement used.

Chemical Compounds ^a^		Mineralogical Composition ^b^	
Oxides	(%)	Compound	(%)
Al_2_O_3_	4.41	C_3_S	55.69
SiO_2_	18.66	C_2_S	11.56
Fe_2_O_3_	2.94	C_3_A	6.72
CaO	60.30	C_4_AF	8.94
MgO	3.93		
SO_3_	2.74		
CaO-free	1.54		
Na_2_O + K_2_O	0.66		
Insoluble residue	0.82		
Loss of ignition	3.44		

^a^ The oxide values presented are the monthly average data issued by the manufacturer. ^b^ The mineralogical composition was obtained through Bogue’s equation.

**Table 2:** Physical properties of the Portland cement used.

Expansion (mm)		0.1
Setting time (min.)	Start	170
	End	210
Surface area Blaine (cm·g^−1^)		4348 ± 52
Compressive strength (MPa)	1 day	25.7
	3 days	40.1
	7 days	45.6
Specific gravity (g·cm^−1^)		3.06

**Table 3:** Main characteristics of the silane used.

Properties	VTES	AEAPTMS	MCPTMS
Purity (%)	>97	>97	>97
Density (g·mL^−1^)	0.903	1.019	1.045
Manufacturer	Gelest (EUA)	Gelest (EUA)	Gelest (EUA)
Hydrolyzable group	Ethoxy	Methoxy	Methoxy
Functional group	Vinil	Amino	Acrilic
Chemical formula	C_8_H_18_O_3_Si	C_8_H_22_N_2_O_3_Si	C_10_H_20_O_5_Si
Molar mass (g·mol^−1^)	190.31	222.35	248.35
Molecular structure	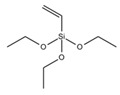	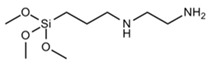	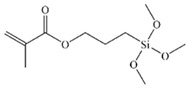

**Table 4:** Paste compositions.

Sample	Cement	Water	Silane	Admixture
REF	1	0.186	0	0.010
VTES 20%			0.002	0.008
VTES 40%			0.004	0.006
AEAPTMS 20%			0.002	0.008
AEAPTMS 40%			0.004	0.006
MCPTMS 20%			0.002	0.008
MCPTMS 40%			0.004	0.006

## Data Availability

The original contributions presented in this study are included in the article. Further inquiries can be directed to the corresponding author.
